# Supplementary material for: Assessing the Implementation of Pharmacogenomic Panel-Testing in Primary Care in the Netherlands Utilizing a Theoretical Framework
Source: J Clin Med. 2020 Mar 17;9(3):814. doi: 10.3390/jcm9030814 (PMC7141350; doi:10.3390/jcm9030814)
Supplement: Supplementary file 1 [file jcm-09-00814-s001.pdf]

Additional file 1: List of 12 genes and interacting 39 drugs which are implemented within the PREPARE study.

Genes included in the PREPARE PGx panel (*n* = 12)	Interacting drugs for which initiation enables study participation (*n* = 39)
CYP2B6	Efavirenz
CYP2C9	Acenocoumarol, phenprocoumon, warfarin, phenytoin,
CYP2C19	Clopidogrel, citalopram, escitalopram, sertraline, voriconazole,
CYP2D6	Flecainide, propafenone, codeine, tramadol, tamoxifen, paroxetine, venlafaxine, amitriptyline, clomipramine, doxepin, imipramine, nortriptyline, metoprolol, aripiprazole, haloperidol, pimozide, zuclopenthixol,
CYP3A5	Tacrolimus
DPYD	Capecitabine, fluorouracil, tegafur,
F5	oestrogen containing oral contraceptives
HLA-B (57:01)	Flucloxacillin
SLCO1B1	Atorvastatin, simvastatin
TPMT	Azathioprine, mercaptopurine, thioguanine
UGT1A1	Irinotecan,
VKORC1	Acenocoumarol, phenprocoumon, warfarin

Additional file 2: PGx recommendation specific survey following receipt of an actionable PGx recommendation (translated from the original in Dutch)

When the drug-gene interaction was actionable:

1. Did you discuss this pharmacogenetic recommendation with the treating physician of this patient?
    a. Yes
    b. No
2. Who discussed the results of the PGx recommendation with the patient?
    a. The pharmacist
    b. The pharmacy technician
    c. The treating physician
    d. Result was to be reported at time of interview
3. Do you support the pharmacotherapy change agreed upon as a result of the PGx results?
    a. Yes
    b. No
4. Why do you not support this? (*when question 3 was answered with b)*

a. *Open text*

5. Approximately how much time did you spend on handling this recommendation?

…… minutes

Additional file 3: Pharmacist survey regarding demographics and perceptions on PGx (translated from the original in Dutch)

Part 1. Pharmacist

1. Do you generally manage the pharmacogenetic recommendation yourself?
    a. Yes
    b. No
    c. I don't know
2. What is your age?
3. What is your gender?
    a. Male
    b. Female
    c. Other / I don't know
4. How many years of work experience do you have as a community pharmacist?
5. What is your function in the pharmacy? (select one)
    a. Managing Pharmacist
    b. Supporting Pharmacist
    c. Community pharmacist specialist in training
    d. Other
    e. I don't know
6. How would you estimate your knowledge of pharmacogenetics?
    a. 1 – I don't know anything about it
    b. 2
    c. 3
    d. 4
    e. 5 – I know something about it
7. To what extent do you think preventive pharmacogenetic testing is effective?
    a. 1 – Not at all effective
    b. 2

    c. 3
    d. 4
    e. 5 – Very effective

8. Did you complete the U-PGx elearning?
    a. No
    b. Yes, partly (please fill in question 9)
    c. Yes, completely
    d. I don’t know

9. When partly, what section of the e-learning did you complete?
    a. The information videos
    b. The case reports
    c. Parts of both sections

Part 2. Pharmacy

1. How many patients does your pharmacy serve?
    a. < 5,000
    b. 5,000 – 7,500
    c. 7,500 – 10,000
    d. > 10,000
    e. I don’t know

2. Is the pharmacy part of a chain or partnership with other pharmacies?
    a. No, completely independent
    b. Yes, a chain
    c. Yes, a franchise
    d. Yes, a pharmacy group
    e. I don’t know

3. How many pharmacists work in your pharmacy?
    a. ≤ 1 FTE
    b. > 1 en ≤ 2 FTE
    c. > 2 en ≤ 3 FTE
    d. > 3 FTE
    e. I don’t know

4. Is the pharmacy located in a health center?

a. Yes
b. No
c. I don't know

Additional file 4: Interview template semi-structured interview (translated from the original in Dutch)

- □ To date you have received … actionable recommendations, of which … were adhered to, is this correct?
- □ How do you generally handle the receipt and implementation of a recommendation?
- □ What do you think about how the handling process generally works? (from receipt of the recommendation until the implementation – so discussion with the treating physician, looking up information etc.)
  - ➔ Ask subsequent questions about mentioned parts of the process

Knowledge	Do you think you have enough knowledge about pharmacogenetics?<br>Would you like to learn more about it? How?<br>Are you aware of the evidence for pharmacogenetics and do you think that is enough?<br>Do you know what is expected of you when handling the recommendation?
Skills	Do you know how to apply for a pharmacogenetic test outside the context of the study?
Professional role	Whose responsibility is it to handle the recommendation?<br>And to do a possible follow up?
Belief in capability	How confident are you in handling the recommendation?<br>So, for example, in consultation with the doctor, or passing it on to a patient?
Optimism	What is your attitude towards pharmacogenetics in general? Are you optimistic about it?
Belief in consequences	To what extent is following the recommendation pleasant or interesting for yourself?<br>Does following the recommendation give you a satisfied feeling?<br>Do you think pharmacogenetics and following the guidelines have a positive effect on the treatment of patients?<br>Do you think the patients appreciate it?<br>What do you think this does with your relationship with the doctor? Does it give better cooperation or not?
Intentions	How do you think things will go after the study ends?<br>Are you going to also apply pharmacogenetic testing outside the context of the study?
Priority / Time	How often does something else on your agenda have a higher priority or urgency than handling a pharmacogenetic recommendation?<br>Which part of handling recommendation takes the most time? How do you think this could be more efficient?
Characteristics of the innovation	Can pharmacogenetics fit into your daily practice? How do you do this?

	You have indicated that handling the recommendation takes on average ... minutes, what do you think of this time spent?
Socio-political context	Is there enough support for pharmacogenetics from the government and, for example, insurance companies?<br>Do you feel supported by the chain/pharmacy group/to participate in this study and to apply pharmacogenetic testing outside of it?
Patients	What is your impression of patients' opinions on pharmacogenetic testing?<br>Do you feel that patients still need something extra?
Sources	Do you know where you can go for information on pharmacogenetics?<br>What do you think of the possibilities within the AIS with regard to pharmacogenetics?
Social and professional influences	Do you ever talk to pharmacists about pharmacogenetics? Do they agree with you or do they have a completely different opinion? How does that affect your opinion?<br>Does it make a difference with which doctor you should take the recommendation? Does that influence the outcome of the intervention? How would you like to see it?
Emotions	Does the handling of the pharmacogenetic recommendation ever cause emotions in you? For example frustration, nervousness or optimism and a good feeling? In which cases is that, why is that and how could it be prevented?
Behavior	If you were to apply a pharmacogenetic test outside the study, in which situation would you do that? And in which patient groups?<br>How do you solve it if a patient or doctor does not want to cooperate with the intervention?<br>How do you solve it if there is not enough time for pharmacogenetic testing?
Source of behavior	Is pharmacogenetic testing and recommendation handling in your daily workflow? Do you think this is also the case outside the context of the study?<br>Do you think this will ever be the case, what would make it happen?

Completion:

- □ What are the reasons why you would (possibly) not follow the recommendation?
- □ When you look back on everything that we have now discussed, what are the most important points for you regarding the implementation of pharmacogenetics?
- □ Ask more specifically for facilitators and barriers
- □ Thank for time and participation
